# Formulation and optimisation of lamivudine‐loaded Eudragit^®^ S 100 polymer‐coated pectin microspheres for colon‐specific delivery

**DOI:** 10.1049/nbt2.12010

**Published:** 2021-02-02

**Authors:** Satheesh Vilas, Sivasudha Thilagar

**Affiliations:** ^1^ Department of Environmental Biotechnology Bharathidasan University Tiruchirappalli Tamil Nadu India

## Abstract

This investigation is to find a prolonged or delayed drug release system, exclusively for the treatment of hepatitis‐B to reduce the side effects, which arise when conventional solid dose forms are administered. To pursue this goal, lamivudine‐loaded Eudragit‐coated pectin microspheres have been formulated employing water/oil (W/O) emulsion evaporation strategy. The formulation was optimised using a 3^4^ factorial design. A drug to polymer ratio of 1:2, the surfactant of 1 ml, the volume of 50 ml of processing medium with a stirring speed of 2500 rpm were found to be the optimal parameters to obtain the lamivudine‐loaded Eudragit‐coated pectin microspheres formulation with a high drug entrapment efficiency of 89.44% ± 1.44%. The in vitro release kinetics of lamivudine was a suitable fit to the Higuchi model, indicating a diffusion‐controlled release with anomalous transport. The obtained microspheres were then subjected to different characterisation studies, including scanning electron microscopy (SEM), Fourier transform infrared spectroscopy (FTIR), differential scanning calorimetry (DSC), and X‐ray diffraction (XRD). The results of this study clearly indicate that Eudragit‐coated pectin microspheres could be the promising controlled release carriers for colon‐specific delivery of lamivudine in the presence of rat cecal content.

## 1 INTRODUCTION

Precise colon drug delivery systems (CDDS) have been developed as site‐specific delivery systems for several therapeutic agents to achieve local and systemic effects [1,2]. CDDS could be of extra value if systemic absorption delays are needed [3]. Chronic hepatitis B remains a major problem worldwide as a result of the high prevalence (300 million) of chronic carriers of the hepatitis B virus (HBV), and the clinical consequences of this disease include liver cirrhosis and hepatocellular carcinoma [4]. At present, there are two therapies for the treatment of chronic hepatitis B: alpha‐interferon (α‐IFN) [5] and the recently approved lamivudine [6,7]. Out of these two, the use of interferon is restricted because of the high cost, side effects, and the risk of liver failure during a flare of hepatitis among patients with cirrhosis. These restrictions do not apply to oral antiviral agents such as lamivudine, which can produce marked viral suppression and reduction of hepatic necro‐inflammatory activity [6,8]. Lamivudine is a nucleoside analogue with potent inhibitory effects on the DNA polymerase activity of HBV [9]. The main concern in lamivudine therapy is that lamivudine‐resistant HBV mutants may emerge after 6–9 months of therapy [10]. The oral administration of lamivudine exhibits side effects in the gastrointestinal tract (GIT) and central nervous system (CNS). Thrombocytopenia, paraesthesias, anorexia, nausea, abdominal cramps, depressive disorders, cough, and skin rashes have also been reported as possible adverse reactions [11]. This necessitates the investigation of alternative dosage forms by which targeting or specificity could be achieved so that the side effects mentioned earlier and other disadvantages inherent to the conventional dosage forms can be avoided. Colon drug delivery is a thrust area of research nowadays, not only because of its applications in the treatment of local diseases but also because of the prospect of its delivering different active pharmaceutical substances. To achieve this colon‐specificity, it is necessary to generate a dosage form that is not absorbed in the upper part of the GIT but released at the optimum location. Oral targeting strategies to the colon include covalent bonding of the drug with the carrier, pH‐responsive polymer coating, timed‐release dosage forms, bio‐adhesive carriers, carriers degraded by colonic bacteria, and osmotic drug delivery systems [12]. Plant polysaccharides have also been investigated in the effective formulation of colon targeted release systems. The main advantage of using such polysaccharides comes from the fact that they remain unaffected in the presence of GI enzymes. Owing to the advantages, pectin polysaccharide has been exploited in the formulation of the colon delivery dosage form of lamivudine. To overcome premature drug release, these natural polysaccharides are further modified either chemically or by being mixed with hydrophobic polymers as these polymers show good film‐forming properties and resistance against pancreatic enzymes. But, natural polysaccharides undergo degradation because of pectin enzymes from bacteria. One disadvantage of pectin is its solubility. This drawback can be overcome by changing its degree of methylation or by synthesising it as calcium pectinate [13,14]. Response surface methodology (RSM) is useful in simultaneously analysing the variables when interactions of such variables are complicated. RSM was adopted to optimise the parameters in the generation of microspheres [15]. Furthermore, it provides the optimum level of experimental factors required for a given response [16] and defines the interactions between factors and avoids unnecessarily numerous runs [17]. Moreover, to get the selective release of the drug only in the lower part of the intestine, the formulation should incorporate materials that will assist in pH‐dependent release. One such material is Eudragit, which normally does not dissolve in the low pH in the stomach or upper intestine but dissolves in the higher pH of the lower intestine. With this, microspheres have been designed for colon‐specific delivery of lamivudine using the natural polysaccharide pectin and by employing pH‐sensitive Eudragit S100 polymer for the treatment of hepatitis‐B.

## 2 MATERIALS AND METHODS

### 2.1 Material

Eudragit S100 and lamivudine were gift samples from Micro Labs Ltd., Peenya (Bangalore, India). Pectin was purchased from Sisco Research Labs Pvt. Ltd. (Mumbai, India). Span 85 and acetone were purchased from S.D Fine Chem Ltd. (Mumbai, India). Liquid paraffin was obtained from Rankem (Ranbaxy) (New Delhi, India). Ethanol was purchased from Merck Chemicals Pvt. Ltd. (Mumbai, India). Isooctane was obtained from Chemlabs (Bangalore, India), and *n*‐hexane was purchased from Samsung Fine Chemicals (Mumbai, India). IKA Ultra instruments Digital Mechanical Overhead Stirrer, Mumbai, Maharashtra, India.

### 2.2 Methods

#### 2.2.1 Preparation of eudragit‐coated pectin microspheres

Emulsion dehydration technique was used to prepare the pectin microspheres. A weighed volume of medication [lamivudine (500 mg)] and polymer [pectin (1 g)] was dissolved in 20 ml of distilled water for this reason and stirred overnight to fully solubilise them. This polymer‐drug solution was distributed in 50 ml of iso‐octane containing Span 85 and stirred continuously for 1 h using the IKA 3593001 RW 20 Digital Mechanical Overhead Stirrer to obtain stable water/oil (W/O) emulsion. This emulsion was then rapidly cooled to 15°C and dehydrated using 50 ml of acetone and was then maintained at 25°C for 30 min under mechanical agitation to allow for complete solvent evaporation. Twenty‐nine formulations were generated using different concentrations of surfactant, a drug to polymer ratio, the volume of processing medium, and stirring speed. The obtained pectin microspheres were freeze‐dried overnight and kept in an airtight container for further studies. Pectin microspheres were coated with Eudragit S100 (ES100) by oil‐in‐oil solvent evaporation method. For this, 50 mg of pectin microspheres were dispersed into 10 ml of coating solution, which was prepared by dissolving 500 mg of ES100 in ethanol and acetone in the ratio of 2:1. This organic phase was then poured into 50 ml of light liquid paraffin containing 2% (*w/v*) of Span 85. This was maintained under agitation (300 rpm) at room temperature for 3 h to allow solvent evaporation. Microspheres were then collected by filtration and washed with *n*‐hexane to remove excess liquid paraffin and were redispersed in distilled water followed by lyophilisation [18].

#### 2.2.2 Experimental design

A 3^4^ Box‐Behnken Design (BBD) was used for the optimisation. The independent variables selected were: drug to polymer (mole ratio) (*x*_1_), surfactant concentration (*x*_2_), the volume of processing medium (*x*_3_), and stirring speed (*x*_4_) with low, medium, and high concentrations(Table 1).

**TABLE 1:** The levels of different process variables in coded and un‐coded form for the drug entrapment efficiency (DEE)

Independent variables	Range and Levels		
	−1	0	+1
Drug polymer ratio, *x*_1_	1:2	1:4	1:6
Surfactant concentration, *x*_2_	1	2	4
Volume of processing medium, *x*_3_	25	50	75
Stirring speed (rpm), *x*_4_	1000	2000	3000

For each factor, the experimental range was selected based on the results of preliminary experiments. The best‐fitting mathematical model was selected based on the comparison of several statistical parameters including the coefficient of variation (CV), the multiple correlation coefficient (*R* ^2^), The adjusted multiple coefficient of correlation (adjusted *R* ^2^) and the expected square residual sum (PRESS) given by Design‐Expert.

#### 2.2.3 Scanning electron microscopy

The size, shape, and surface morphology of pectin microspheres and Eudragit‐coated pectin microspheres were investigated using scanning electron microscopy (SEM) (JEOL Version 1.1 JSM 6360, Japan) operating at 20 kV. For the analysis, the samples were mounted on aluminum studs using double‐adhesive tapes and vacuum‐coated with platinum using JEOL JFC‐1600 Auto Fine Coater to render the samples electrically conductive.

#### 2.2.4 Fourier transform infrared spectroscopy

The Fourier transform infrared spectroscopy (FTIR) spectra were recorded in solid phase on an Avatar 320‐FTIR (UK) spectrometer in the region of 4000–400 cm^‐1^. Samples were ground with KBr and compressed to make pellets. The following samples were assayed: (1) lamivudine, (2) empty Eudragit‐coated pectin microspheres, (3) physical mixture of pectin, Eudragit S‐100, and lamivudine, and (4) lamivudine‐loaded microspheres.

#### 2.2.5 Differential scanning calorimetry

Differential scanning calorimetry (DSC) measurements were performed with a DSC 6200 thermal analysis system (Seiko Instruments Inc., Tokyo, Japan). Samples (4 mg) were heated from 25°C to 200°C in sealed aluminum pans at a scanning rate of 10°C/min under nitrogen purge with an empty aluminum pan as reference.

#### 2.2.6 Determination of entrapment efficiency

The drug‐loaded microspheres (100 mg) were powdered and suspended in 100 ml of the acetone–ethanol mixture, and 6 ml of acetonitrile was added and vortexed for 5 min. The drug content was determined by measuring the absorbance at 261 nm using a UV–Vis spectrophotometer (Shimadzu, UV‐Vis 1700, Japan). The efficiency of drug trapping was determined with the following equation.

(1) $$\text{Entrapment}\;\text{efficiency}\left(EE\right)(\%)\;=\frac{\text{Weight}\;\text{of}\;\text{Lamivudine}\;\text{in}\;\text{the}\;\text{microspheres}}{\text{Input}\;\text{weight}\;\text{of}\;\text{Lamivudine}}\times 100$$

All the formulations were analysed in triplicate (*n* = 3).

#### 2.2.7 X‐ray diffraction

Samples were subjected to X‐ray diffraction (XRD) by employing a low angle X‐ray diffractometer (Seifert 2002, Germany), where scanning was made from 0° to 60° (2θ) with an increment of 0.02° (2θ).

#### 2.2.8 In vitro drug release

The tests of drug release in vitro were carried out using a dissolution apparatus (USP XXIII) of the paddle type. Drug loaded microspheres were suspended in 450 ml of dissolution medium and were stirred continuously at 100 rpm for 12 h at 37°C. Aliquots of the dissolution medium (2 ml) were withdrawn at predetermined time intervals, and the concentration of the drug was analysed at 261 nm using a UV–Vis spectrophotometer.

#### 2.2.9 In vitro release of Eudragit coated lamivudine loaded pectin microspheres in the presence of rat cecal content

In vitro release of lamivudine from the optimised batch of Eudragit‐coated pectin microspheres was performed in the presence of rat cecal content to measure the biodegradability of pectin by the colonic bacteria. Four albino rats (Wistar strain) of uniform body weight (150–200 g) were maintained on a normal diet and administered with 1 ml of 2% dispersion of pectin in water. This treatment was undertaken for 7 days to induce the enzymes that particularly act on the pectin. After 7 days, rats were sacrificed, and the abdomen was opened. The cecum was located and tied at both ends. Then, it was dissected and transferred into phosphate‐buffered saline (PBS) of pH 6.8 without any delay. The cecal bag was carefully opened, and the contents were immediately weighed, and the required amount was suspended in the simulated intestinal fluid of pH 7.4 to get 2% cecal content. It was used as a simulated colonic fluid to perform the release study. The tests were performed with a steady supply of CO_2_ into the dissolution environment. Studies of drug release for the initial 4 h were performed as mentioned above in simulated GI fluids. The studies were conducted in replicated intestinal fluid containing cecal content of rats after 4 h. At regular intervals, aliquots of samples were extracted and replaced with a new buffer bubbled with CO_2_. The samples were filtered using Whatman filter paper following the determination of drug content.

## 3 RESULTS AND DISCUSSION

### 3.1 Optimisation of the percentage of drug entrapment

Optimisation of the surface response is more desirable than conventional optimisation of a single parameter as it saves time. A total of 29 experiments were conceived and carried out at random (Table 2).

**TABLE 2:** Box‐Behnken design matrix along with predicted and experimental values of percentage drug entrapment efficiency (DEE)

Run no	*x*_1_	*x*_2_	*x*_3_	*x*_4_	% Drug entrapment efficiency	
					Experimental	Predicted
1	0	1	0	‐1	48.52	50.36
2	0	‐1	‐1	0	39.12	39.42
3	0	1	0	1	56.43	57.25
4	‐1	0	0	‐1	36.43	36.47
5	0	0	‐1	‐1	30.21	29.11
6	0	0	0	0	53.98	53.98
7	1	1	0	0	66.32	66.52
8	0	0	1	‐1	47.21	47.52
9	0	0	1	1	45.42	46.5
10	1	0	0	‐1	49.54	48.69
11	‐1	0	‐1	0	36.32	37.54
12	1	0	‐1	0	47.21	48.04
13	0	0	0	0	53.98	53.98
14	0	‐1	1	0	45.32	45.67
15	‐1	‐1	0	0	45.32	45.1
16	‐1	0	1	0	46.32	46.08
17	‐1	0	0	1	47.13	47.42
18	0	1	‐1	0	49.36	48.45
19	0	1	1	0	59.74	58.88
20	0	0	0	0	53.98	53.98
21	0	0	0	0	53.98	53.98
22	0	‐1	0	‐1	37.32	37.09
23	0	0	0	0	53.98	53.98
24	1	0	0	1	56.43	55.82
25	‐1	1	0	0	50.43	49.84
26	1	0	1	0	56.83	56.19
27	0	0	‐1	1	48.54	48.21
28	1	‐1	0	0	47.46	48.53
29	0	‐1	0	1	49.54	48.28

The experimental data obtained have been statistically analysed using version 7.1.6 of the Design Expert trial for variance analysis (ANOVA), and the findings are shown in Table 3.

**TABLE 3:** Analysis of variance (ANOVA) for response surface quadratic model for the percentage drug entrapment efficiency

Source	Sum of squares	D.F.	Mean square	*F* value	*p* value Prob > *F*	
**Model**	1660.64	14	118.62	101.96	<0.0001	Significant
**A‐A**	318.68	1	318.68	273.93	<0.0001	
**B‐B**	370.96	1	370.96	318.87	<0.0001	
**C‐C**	209.00	1	209.00	179.65	<0.0001	
**D‐D**	245.35	1	245.35	210.89	<0.0001	
**AB**	47.27	1	47.27	40.63	<0.0001	
**AC**	0.036	1	0.036	0.031	0.8727	
**AD**	3.63	1	3.63	3.12	0.0992	
**BC**	4.37	1	4.37	3.75	0.0731	
**BD**	4.64	1	4.64	3.99	0.655	
**CD**	101.20	1	101.20	86.99	<0.0001	
**A2**	12.27	1	12.27	10.55	0.0058	
**B2**	0.35	1	0.35	0.30	0.5910	
**C2**	206.55	1	206.55	177.54	<0.0001	
**D2**	196.42	1	196.42	168.84	<0.0001	
**Residual**	16.29	14	1.16			
**Lack of fit**	16.29	10	1.63			
**Pure error**	0.000	4	0.000			
**COI total**	1676.93	28				

The regression model's Analysis of variance (ANOVA) shows that the model is highly significant, as is evident from the measured *F*‐value (101.96) and very low probability value (*P* ≤ 0.0001). The predicted *R* ^2^ of 0.9441 is in reasonable agreement with the adjusted *R* ^2^ of 0.9903. ‘Adequate accuracy' tests the ratio of signal to noise, and a ratio greater than four is optimal. A suitable signal is defined by the ratio of 48.23 obtained. At the same time, the coefficient of variance is comparatively lower (CV = 2.21%), indicating a better precision and reliability of the experiments.

To determine the levels of factors (*x*_1_, *x*_2_, *x*_3_, and *x*_4_) that yield the drug entrapment, mathematical relationships were generated between dependent and independent variables (responses) using Design Expert trial version 7.1.6, following which, the reduced Equation (4) was generated for the observed response *Y* after applying ANOVA [19].

(2) $$\begin{array}{l} Y=53.98+5.15x_{1}+13.40x_{2}+4.17x_{3}+4.52x_{4}+3.44x_{1}x_{2}\\ -0.095x_{1}x_{3}-0.95x_{1}x_{4}+1.04x_{2}x_{3}-1.08x_{2}x_{4}-5.03x_{3}x_{4}-\\ 1.38x_{1}^{2}-0.23x_{2}^{2}-5.64x_{3}^{2}-5.50x_{4}^{2} \end{array}$$

where *Y*_1_ is the drug entrapment (%), *x*_1_ is the drug to polymer ratio, *x*_2_ is the surfactant concentration, *x*_3_ is the volume of processing medium, and *x*_4_ is the stirring speed. The value of the coefficient of regression (*R* ^2^ = 0.9903), which is closer to one, suggests that the correlations are best suited for predicting the values for drug trapping, and that the expected values are similar to the experimental results (Table 2).

**FIGURE 1:**
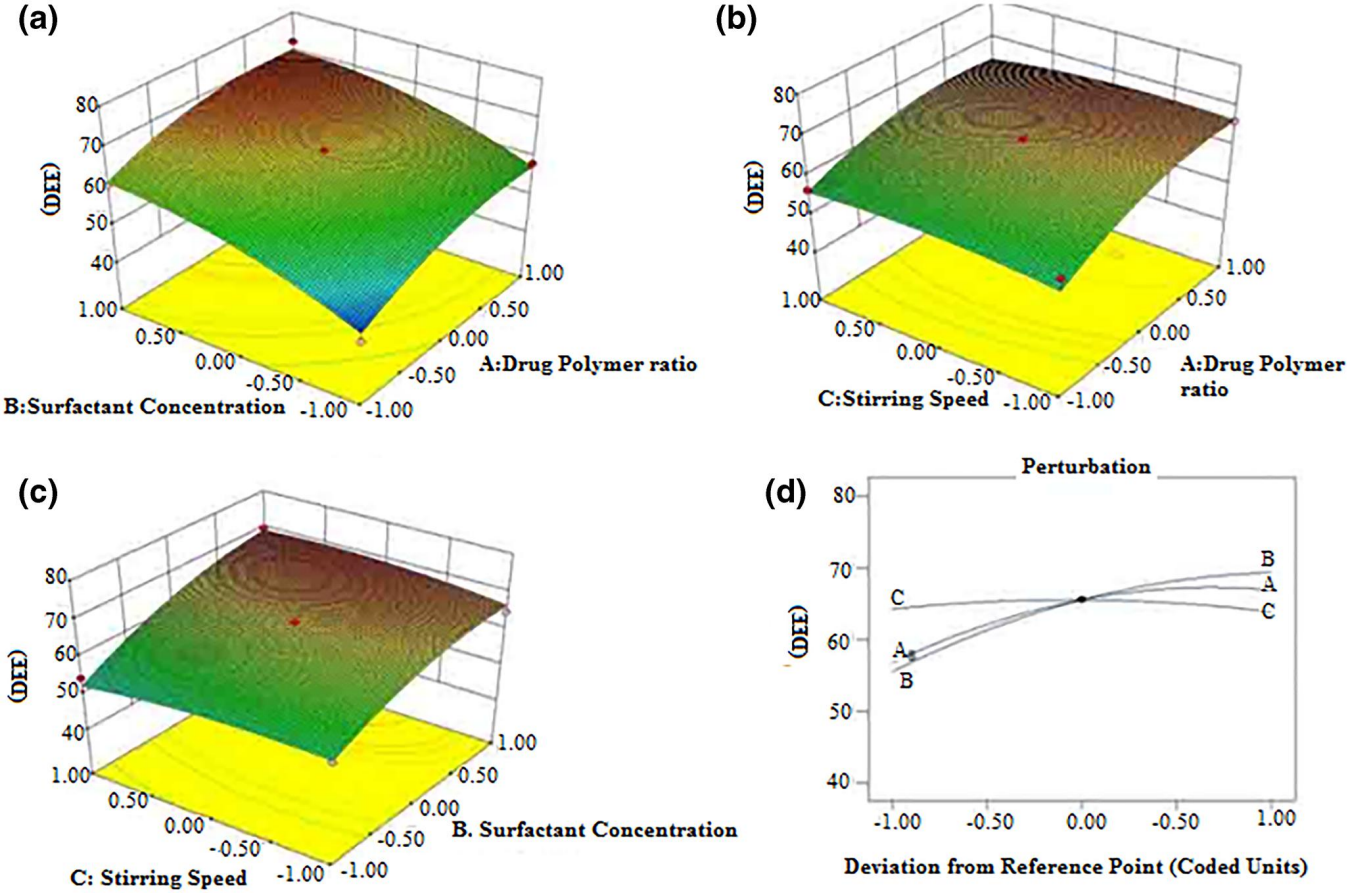
(a–c) Response surface (3D) showing the effect of different drug entrapment parameters (*x*_1_: drug polymer ratio; *x*_2_: surfactant concentration; *x*_3_: volume of processing medium; *x*_4_: string speed) added on the response (DEE)

The response surface curves for drug entrapment efficiency (%) are shown in Figure 1a–c.

Y_1_ and Figure 1d perturbation plot indicating the impact of each of the components on the drug entrapment efficiency (DEE). Drug polymer ratio (*x*_1_), lower the entrapment efficiency when the surfactant concentration increased from 1 ml to 4 ml. Also, a higher volume of processing medium and increased stirring speed resulted in higher entrapment efficiency. DEE (%) for microspheres with different polymer ratios is given in Table 1. The number of combinations of the two test variables is represented by each 3‐D map. The surface contained in the smallest curve of the plot shows the highest percentage of drug entrapment with the other variable held at zero amounts. From the response surface and perturbation plots (Figure 1a–d), it is obvious that surfactant concentration had a significant effect on DEE as compared to other variables. The studies of the 3‐D surface plot also reveal the optimal values of the drug to polymer ratio (1:2), surfactant concentration (1 ml), the volume of processing medium (50 ml), and stirring speed (2500 rpm). The experimental data of three batches prepared within optimum range were very close to the predicted values with low percentage error (Table 4), suggesting that the optimised formulation was reliable and reasonable.

**TABLE 4:** Comparison of experimentally observed response of the optimised microspheres Formulation with that of predicted response

Response parameter	Constraint	Observed value	Predicted value^a^	Error%
DEE%	Maximise	46.28 ± 1.32	49.32	3.2

^a^ Mean ± SD, *n* = 3.

### 3.2 Morphological characterisation of microspheres

**FIGURE 2:**
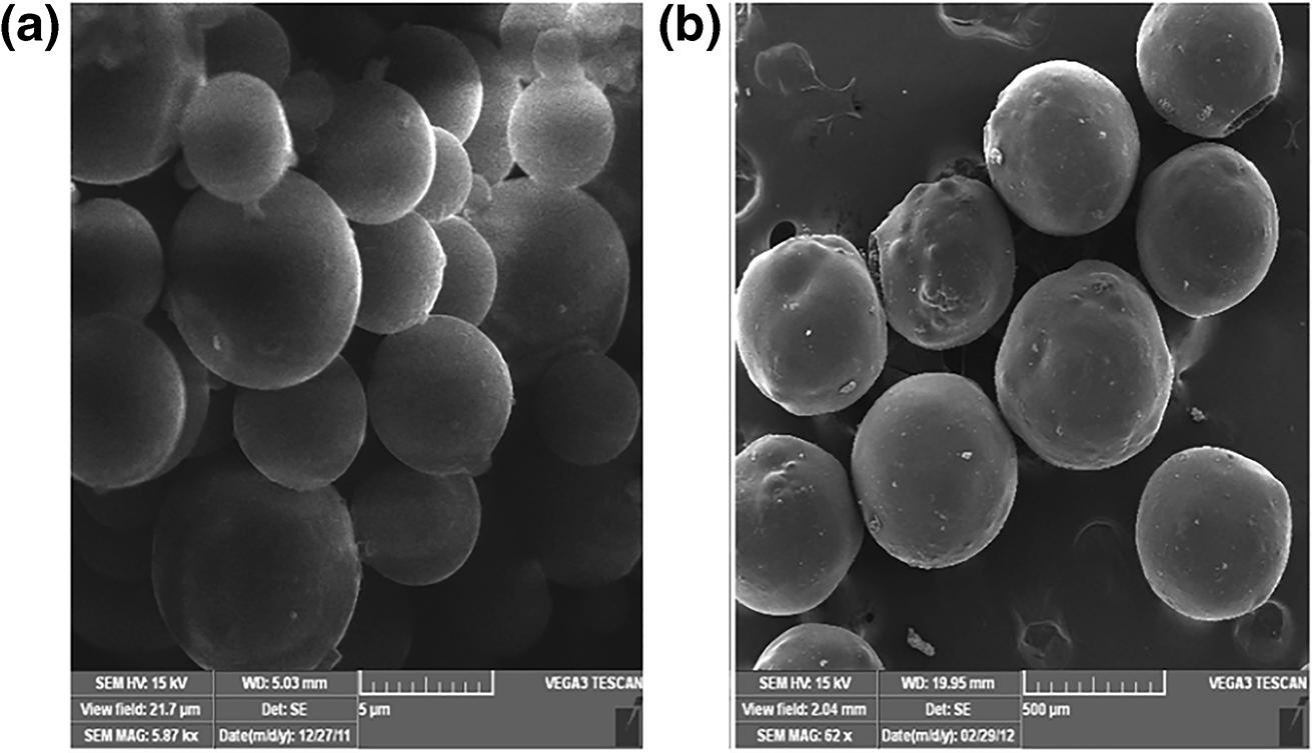
Scanning electron microscopy (SEM) images of (a) uncoated pectin microspheres and (b) Eudragit‐coated pectin microspheres

Figure 2a,b show the pectin‐lamivudine microspheres and Eudragit‐coated lamivudine microspheres, respectively. It could be observed that the microspheres are, in general, smooth and spherical. In this, the polymer was fully saturated, and the diffusion rate of the solvent was minimal, leading to the formation of smooth, sphere‐shaped, individual, and evenly distributed particles with no evidence of collapsed particles. The smooth surface revealed complete removal of the solvent from the formulated micro particles; this ensures good quality of the microsphere.

### 3.3 FTIR analysis

**FIGURE 3:**
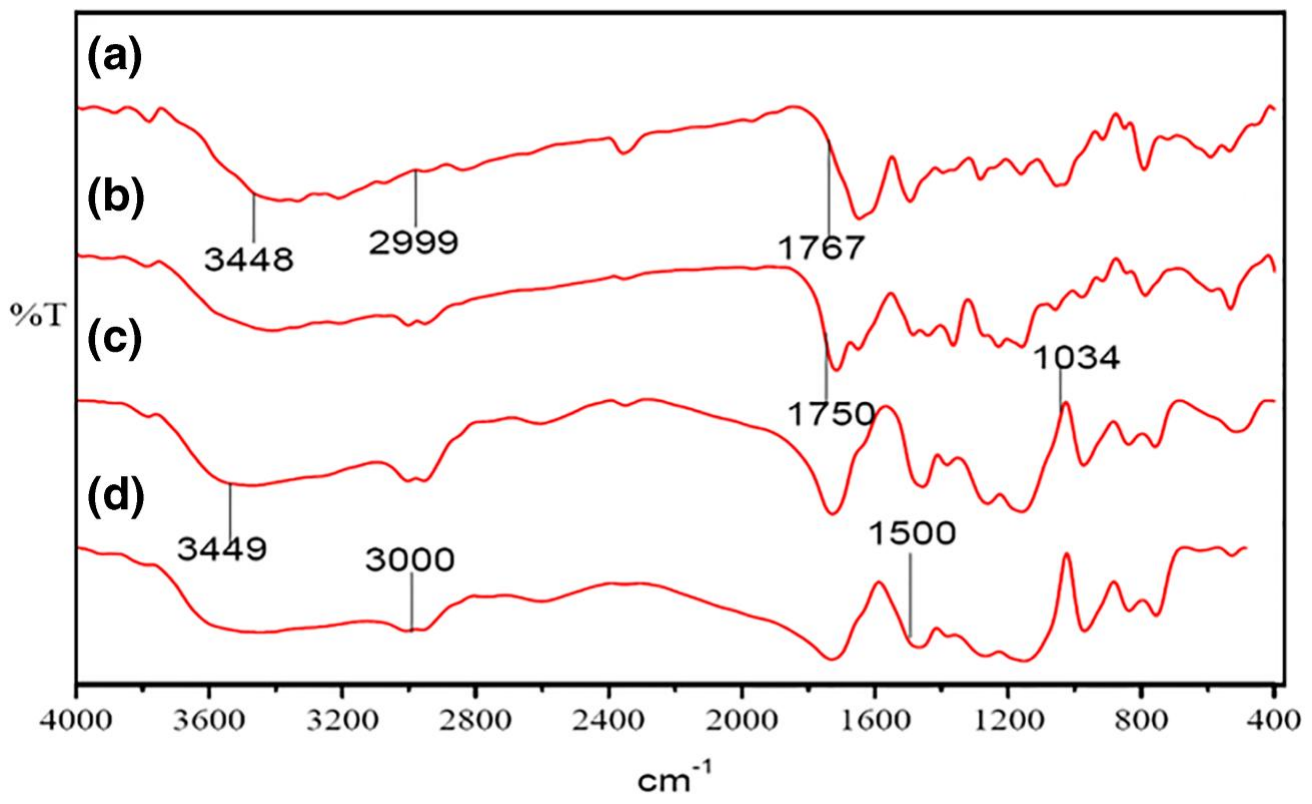
Infrared spectra of: (a) lamivudine, (b) Empty Eudragit‐coated pectin microspheres, (c) physical mixture of pectin, Eudragit‐S100 and lamivudine, (d) lamivudine‐loaded microsphere

FTIR spectral data were used to confirm the chemical stability of lamivudine in polymeric microspheres. FTIR spectra of pure lamivudine, empty Eudragit‐coated pectin microspheres, physical mixture of pectin, Eudragit S‐100 and lamivudine, and lamivudine‐loaded polymeric microspheres are shown in Figure 3.

Figure 3b shows the FTIR spectrum of empty Eudragit‐coated pectin microspheres, where the absorbance is higher at 1750 cm^‐1^ than at 1650 cm^‐1^, and the Eudragit S‐100 polymer showed the characteristic band of the carboxylic groups at 1730 cm^‐1^ [19]. The FTIR spectrum of pure lamivudine drug at 3448, 2999, 1767, and 1458 cm^−1^ was because of the formation of N‐H, O‐H, C=O, and C=N linkages, respectively (Figure 3a) Also, the drug‐loaded microspheres show a broad peak between 1500 and 3000 cm^−1^, which indicates the interaction between the polymer and the drug (Figure 3d). This also confirms that the drug was incorporated into the polymeric microsphere.

### 3.4 Differential scanning calorimetry*****

**FIGURE 4:**
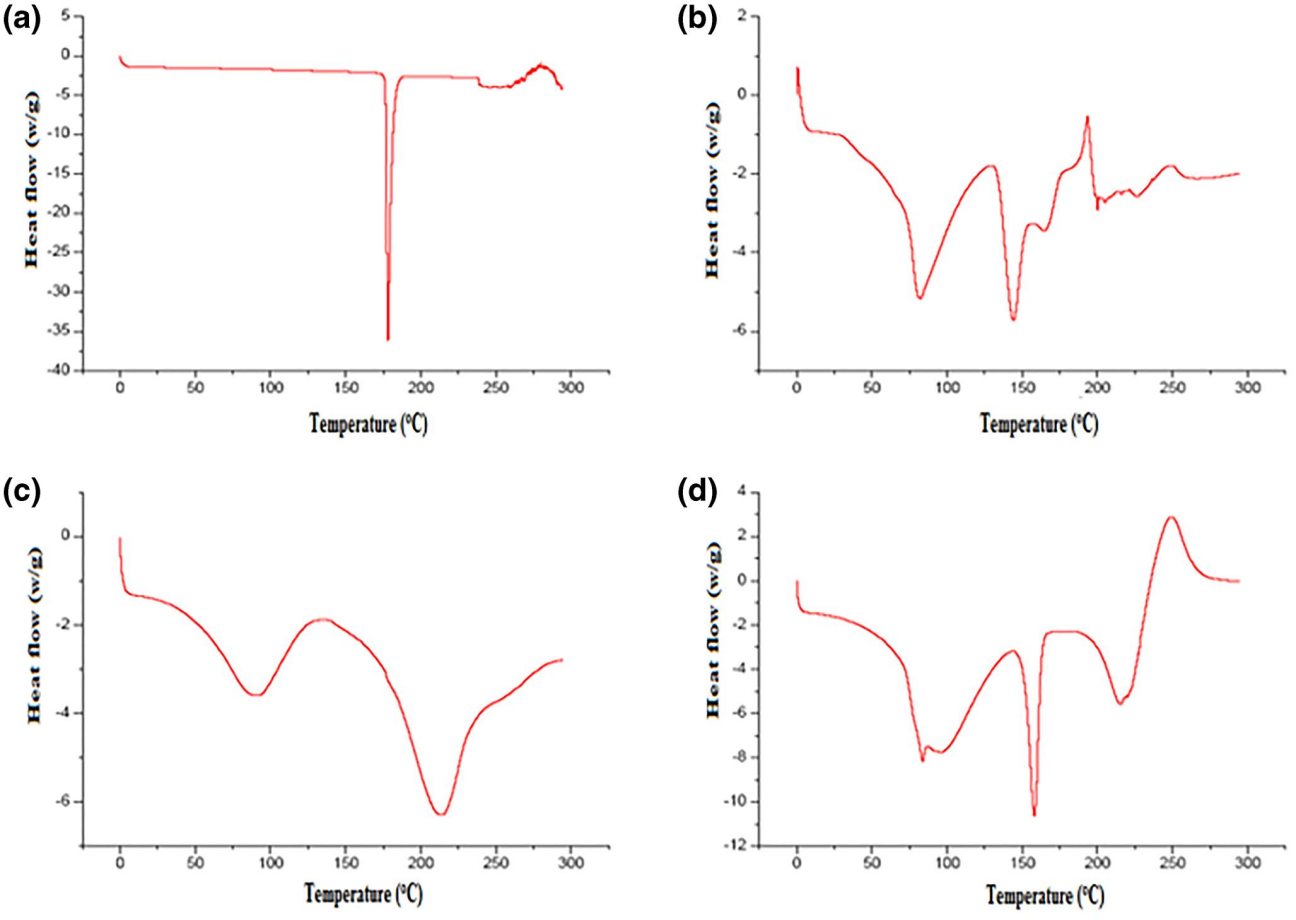
Differential scanning calorimetry (DSC) thermogram of (a) lamivudine pure drug, (b) lamivudine‐loaded microsphere, (c)physical mixture of lamivudine, pectin, and Eudragit(d) empty Eudragit‐coated pectin microspheres

DSC studies were conducted for pure drug, lamivudine‐loaded microspheres, physical mixture of lamivudine, pectin, and Eudragit polymer, and empty Eudragit‐coated pectin microspheres. The pure drug shows a sharp endotherm at 170°C, corresponding to its melting point/transition temperature (Figure 4a), and the polymer shows an endotherm at 149°C (Figure 4c). Drug loaded microspheres show an endotherm at 155°C (Figure 4b). There was no significant change in the formulation's melting endotherms relative to the pure drug, but a slight decrease in the melting temperature was observed, which could be attributed to minor physical and morphological changes in the product and the polymer following the formulation. The difference in endotherms of the polymer and the formulation confirmed that there was no evidence of chemical reaction taking place between the polymer and the drug [20].

### 3.5 X‐ray diffraction analysis

**FIGURE 5:**
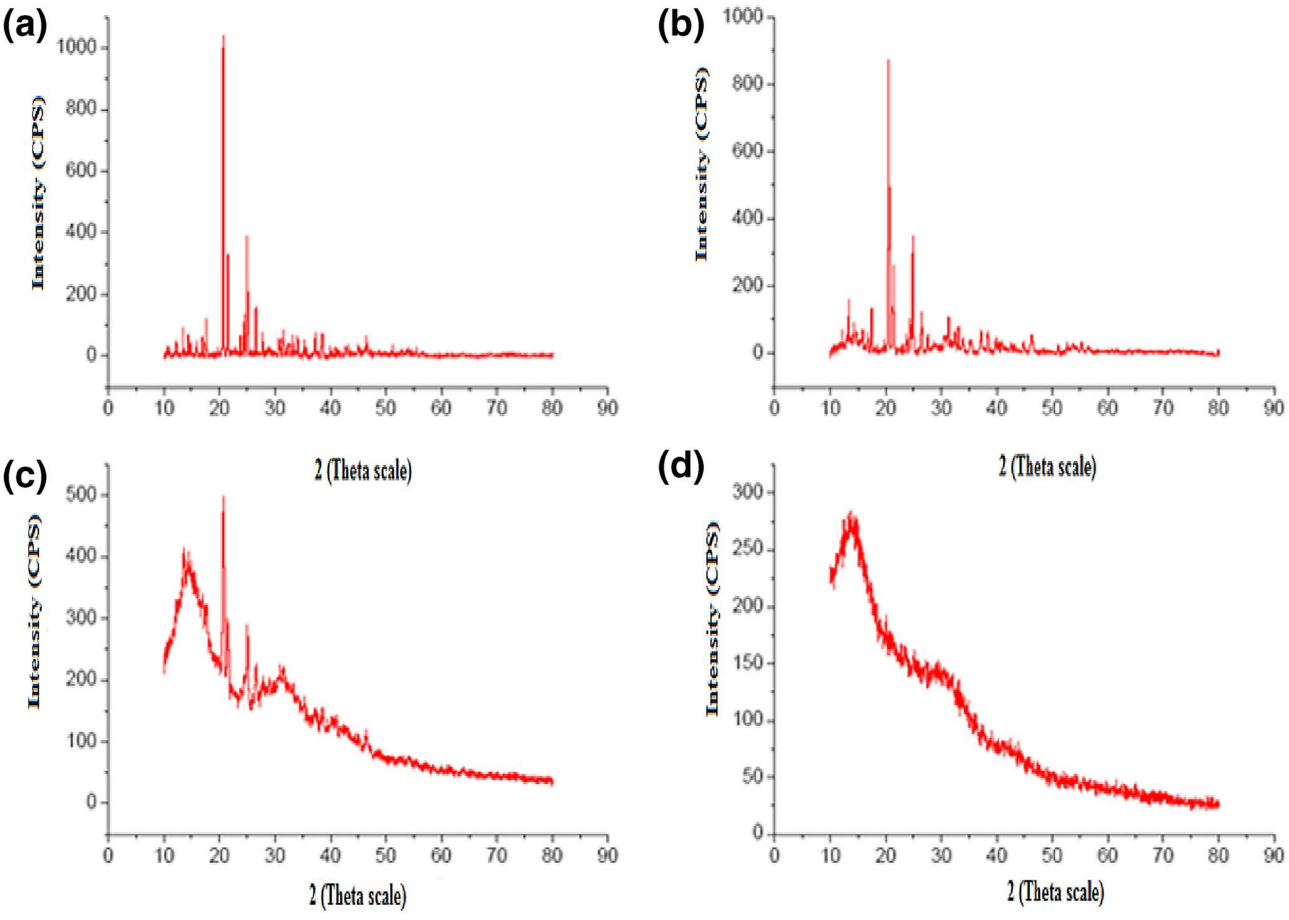
X‐ray diffraction analysis of (a) lamivudine, (b) physical mixture of lamivudine, pectin, and Eudragit (c) empty Eudragit‐coated pectin microspheres, and (d) lamivudine‐loaded microsphere

XRD analysis of the drug, physical mixture of lamivudine, pectin, and Eudragit polymer, empty Eudragit‐coated pectin microspheres, and lamivudine‐loaded microspheres was performed and are shown in Figure 5. Lamivudine shows the characteristic intense peak at 2θ of 22.3°, 24.32°, and 29.18° because of its crystallinity. However, these peaks were not found in lamivudine‐loaded microspheres. Generally, the width of the XRD peaks depends upon the crystallite size. The absence of lamivudine peak in the lamivudine‐loaded microspheres indicates that the lamivudine transformed into an amorphous state at the molecular level when encapsulated in the microspheres [21].

### 3.6 In vitro release of optimal formulation

**FIGURE 6:**
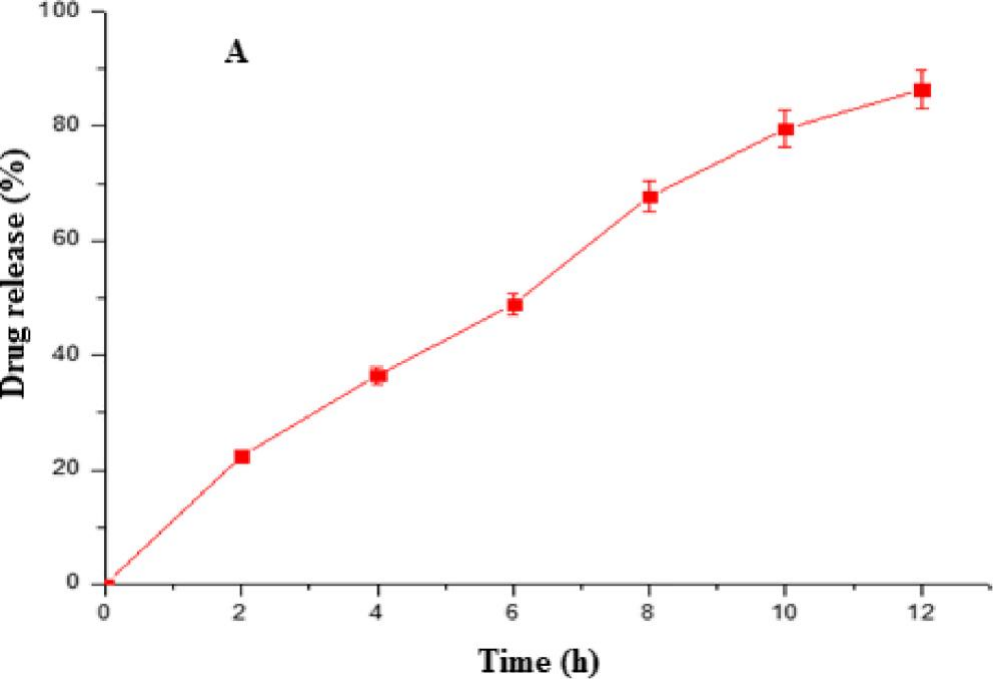
Cumulative mean percent of lamivudine released (a) from dissolution of optimised formulation at pH 7.4 (error bars ± S.D); (*n*=3)

For colon‐specific drug delivery systems to be tested in vitro, the dissolution test method would closely resemble in vivo conditions regarding pH. In vitro release studies were carried out for a known quantity of Eudragit‐coated pectin microspheres using the USP paddle dissolution apparatus at 100 rpm and 37 ± 0.5°C [21]. In order to simulate the pH of the GI tract and the transit time that the colon‐specific delivery system would experience in vivo, various buffers were established for different time periods. The cumulative in vitro drug release was less from the Eudragit‐coated pectin microsphere, which shows that lamivudine was retained efficiently inside the microspheres tested in the buffer systems of pH 1.2 for 2 h with 0.1NHCl. pH was then adjusted to 4.5, and the release study was continued for another 2 h. After 4 h, the pH of the dissolution medium was adjusted to pH 7.4 with 1.0 M NaOH, and the study was continued. From the release data, it was clear that 95% of the drug was retained inside the particle‐matrix at pH 1.2 and pH 4.5 (data not shown). But at pH 7.4, coated microspheres delivered about 20% of the incorporated drug within 2 h (Figure 6a).

**FIGURE 7:**
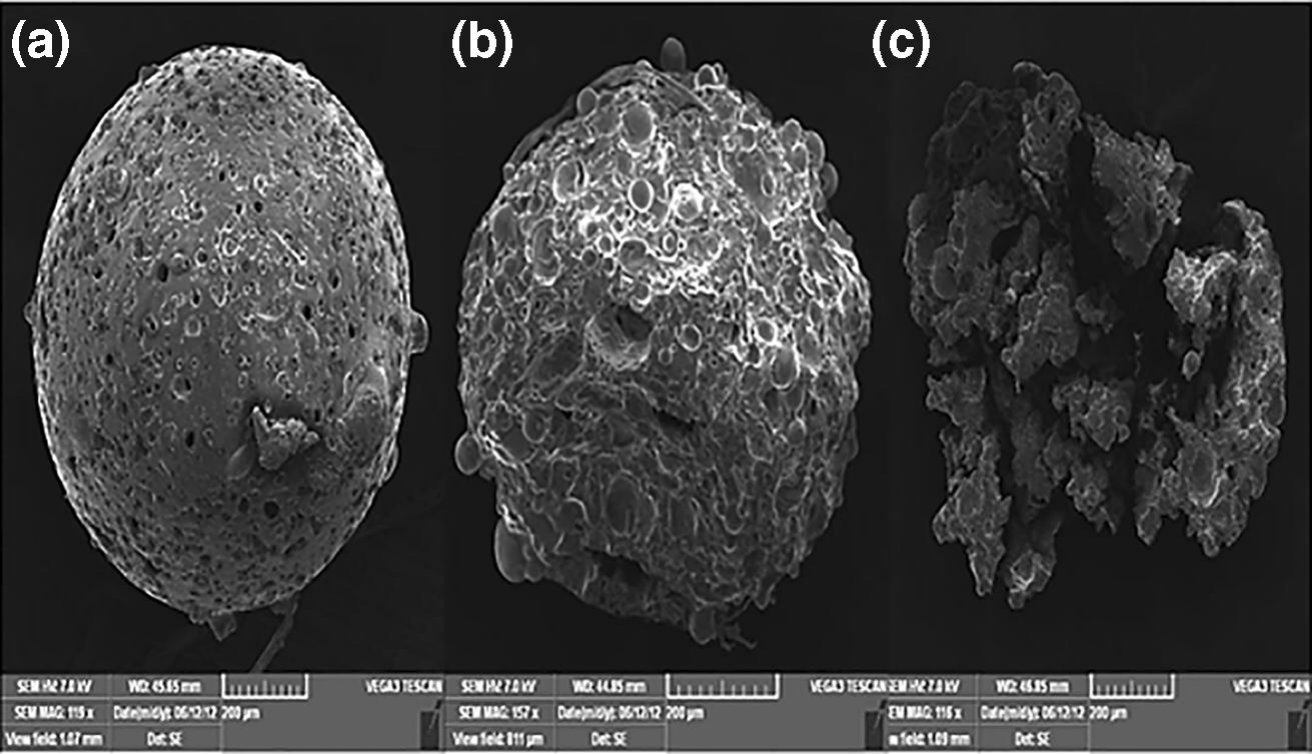
Surface integrity of spherical microspheres during in vitro drug release studies in pH 7.4 (a) at second hour, (b) at sixth hour, and (c) at 12th hour during in vitro drug release of Eudragit‐coated pectin microspheres

It reveals that the drug release from Eudragit coated pectin microspheres was purely pH‐dependent. The changes in the surface integrity of microspheres (spherical shape) during in vitro drug release studies were confirmed by SEM (Figure 7).

The results also show that the Eudragit polymer started dissolving when the pH was 7.4, which indicates that the encapsulated drug can be released in the colonic region [22]. Hence, the colon‐specific drug release can be achieved by using Eudragit coating, and it may be expected in in vivo studies [23]. Using the release data, the following graphs were plotted: (a) log cumulative percentage drug remaining versus time (first‐order); (b) cumulative percentage drug release versus time (zero‐order); (c) cumulative percentage drug release versus square root of time (Higuchi). *R* ^2^ value was calculated to understand the release kinetics. Among them, it was found that the zero‐order models showed a high *R* ^2^ value, that is, 0.9873, indicating that the release of the drug followed zero‐order release kinetics. Similarly, to understand the mechanism of drug release (*d*), the Korsmeyer‐Peppas equation was applied, and good linearity (*R* ^2^ = 0.8187) was observed. The release exponent ‘*n*' was found to be 0.5556. As the value of ‘*n*' lies between >0.43 and <0.85, it indicates that the drug release was following anomalous transport (Non‐Fickian) [24,25], which specifies that the drug release was controlled both by diffusion and erosion mechanisms. The release kinetics results are shown in Electronic Supplementary Material I.

### 3.7 In vitro drug release study in the presence of rat cecal content

**FIGURE 8:**
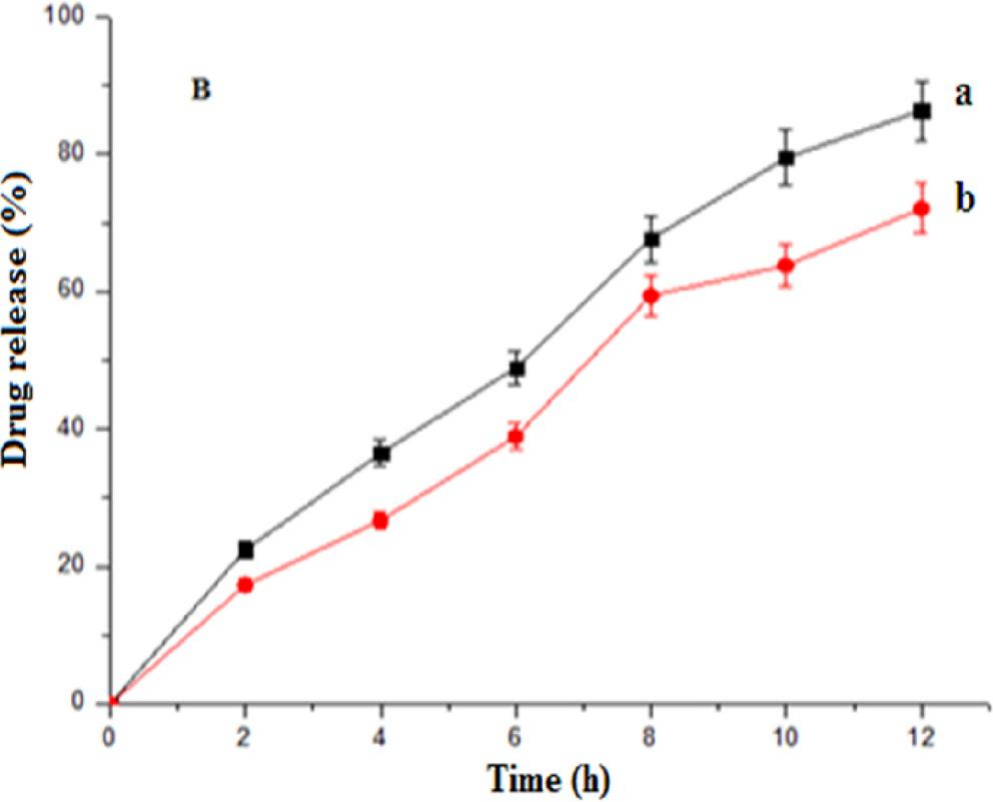
Percentage lamivudine cumulative release (b) from Eudragit‐coated pectin microspheres (a) with rat cecal content and (b) without rat cecal content (Error bars ±S.D); (*n*=3)

The in vitro release of optimised Eudragit‐coated microspheres in the presence of 2% rat cecal content (a) at pH 7.4 showed a faster drug release at different periods when compared with the release study conducted without rat cecal content (b) (Figure 8). This finding could be attributed to various anaerobic bacteria that are present in cecal content and are, thus, responsible for the digestion/degradation of pectin, which in turn releases the drug from microspheres [26].

## 4 CONCLUSION

Pectin microspheres of lamivudine were successfully prepared by the emulsion dehydration technique using different ratios of the polymer. This method of preparing a solution or suspension of pectin and lamivudine is comparatively easier than other techniques. Our study results suggest that the transmission of Eudragit‐coated pectin‐lamivudine microspheres to the colonic area has great scope. This demonstrates the use of Box‐Behnken Model in optimising formulations for the microsphere. The derived polynomial equation and 3‐D surface plot aid in predicting the values of selected independent variables for the generation of optimum microsphere formulations with the desired properties. The SEM, FT‐IR, DSC, and XRD permitted a structural analysis where the molecular dispersion of the drug inside the polymeric microsphere matrix, which allowed efficient drug incorporation and retention, was observed. Hence, the formulated Eudragit‐coated pectin‐lamivudine microspheres could be used for the efficient treatment of hepatitis B Accordingly, the next step of this investigation has been planned to optimise the parameters that influence the efficacy and bio‐availability in vivo.
